# Dengue infection elicits skin tissue-resident and circulating CD8^+^ T cells associated with protection from hospitalization

**DOI:** 10.1126/sciadv.aea7987

**Published:** 2026-03-20

**Authors:** Noor Zayanah Hamis, Justin SG Ooi, Ka-Wai Cheung, Valerie Chew, Michaela Gregorova, Eugenia Ziying Ong, Kuan Rong Chan, Tun-Linn Thein, Yee-Sin Leo, David Chien Boon Lye, Eng Eong Ooi, Laura Rivino

**Affiliations:** ^1^ Emerging Infectious Diseases Programme, Duke-NUS Medical School, Singapore, Singapore.; ^2^ School of Biochemistry and Cellular and Molecular Medicine, University of Bristol, Bristol, UK.; ^3^ Viral Research and Experimental Medicine Centre, SingHealth Duke-NUS Academic Medical Centre, Singapore, Singapore.; ^4^ National Centre for Infectious Diseases, Singapore, Singapore.; ^5^ Tan Tock Seng Hospital, Singapore, Singapore.; ^6^ Yong Loo Lin School of Medicine, National University of Singapore, Singapore, Singapore.; ^7^ Lee Kong Chian School of Medicine, Nanyang Technological University, Singapore, Singapore.; ^8^ Communicable Diseases Agency, Singapore, Singapore.

## Abstract

Dengue is spreading globally, and there is urgent need to define immune correlates of protection for this disease. Dengue infection first occurs in the skin following the bite of an infected mosquito; however, knowledge of host immune responses within this site remains sparse. We investigated the phenotypic, functional, and transcriptional profiles of skin and blood T cells in 73 patients with dengue and 10 healthy volunteers. We show that the skin T cell compartment undergoes marked reshaping and is strongly enriched with proliferating CD4^+^ and CD8^+^ T cells compared with the blood of patients. Activated skin CD8^+^ T cells expressed a core transcriptional signature of tissue-resident memory T (T_RM_) cells, supporting their differentiation to the T_RM_ cell lineage during infection. The magnitude of skin and blood CD8^+^ T cell responses were associated with protection from hospitalization in this cohort. These data support a protective role of skin-resident and circulating CD8^+^ T cells in dengue and warrant evaluation of vaccination strategies inducing skin T_RM_ cells to enhance protective immunity.

## Teaser

Dengue infection induces marked reshaping of the skin T cell compartment and elicits skin tissue-resident CD8^+^ T cells.

## INTRODUCTION

Dengue is caused by dengue virus (DENV), a mosquito-borne flavivirus estimated to infect 390 million people, causing 300,000 severe dengue cases and 20,000 deaths per year (*1*). Urbanization and human mobility, both of which are now further complicated by climate change, have driven a rapid ~10-fold increase in dengue cases in the past 20 years (*2*). DENV cocirculates as four genetically distinct infectious serotypes (DENV 1-4); infection with any DENV serotype may be asymptomatic or cause a range of clinical manifestations from a self-limiting febrile illness to severe dengue characterized by hypovolemic shock from uncontrolled plasma leakage, internal hemorrhage, and organ dysfunction (*3*). The pathogenesis of dengue is multifactorial and poorly understood, although altered host immune responses are believed to play key roles (*3*, *4*). No licensed therapy exists for dengue. There are two licensed dengue vaccines that show unbalanced protection toward DENV1-4 despite their ability to elicit neutralizing antibodies against all four DENV serotypes, thus highlighting our incomplete understanding of the immune correlates of protection for dengue (*5*). In particular, the contribution of T cells for protective immunity to DENV remains unclear. To date, studies have focused on analyses of T cells in the blood of patients with dengue, which may not be representative of T cell responses occurring within tissues where most of T cell responses occur during infection.

T cell responses initiate in the skin following the bite of a DENV-infected *Aedes* mosquito. We and others have shown that T cell priming in dengue occurs in the skin/skin-draining lymph nodes (*6*), where DENV-specific T cells acquire expression of the skin-homing receptor cutaneous leukocyte-associated antigen (CLA) (*7*). CLA binds to E-selectin (CD62E) expressed on dermal endothelial cells and facilitates T cell homing to the skin (*8*). It remains unknown whether DENV-specific T cells in the skin differentiate into tissue-resident memory T (T_RM_) cells and whether these play a role in protective immunity to dengue.

T_RM_ cells were identified a decade ago as specialized T cell subsets located within tissues, which display superior protective efficacy toward pathogens compared with their circulating counterparts (*9*, *10*). T_RM_ cells clear virus-infected cells through release of cytotoxic granules and cytokines/chemokines, with the latter also acting as signals to recruit other leukocytes to the tissue (*11*, *12*). In mice, T_RM_ cells were shown to have a key protective role against herpes simplex virus in the skin and influenza and respiratory syncytial viruses (RSVs) in the lungs, with dispensable roles of circulating T cells (*13*, *14*). The role of T_RM_ cells in human infection remains less clear due to the difficulties in accessing the human tissues where these cells reside. T_RM_ cells were detected in human acute and chronic viral infections (influenza, RSV, hepatitis B, and HIVs) (*14*–*16*), with some studies reporting an association of T_RM_ cell frequencies with enhanced viral control (*17*). Understanding the contribution of T_RM_ cells to protective immunity is critical for evaluation of vaccine delivery routes that could improve vaccine efficacy by boosting generation of protective T cell subsets.

In this study, using a skin-blister induction model that we established for dengue (*7*), we investigated the phenotypic, functional, and transcriptional signatures of T cells in matched skin and blood samples from 73 adult patients with dengue and 10 healthy volunteers. We found that CD4^+^ and CD8^+^ T cell responses to DENV infection were highly enriched in the skin compared with the matched blood compartment. Activated CD8^+^ T cells in the skin displayed phenotypic and transcriptional features of T_RM_ cells including expression of the core gene signature of human T_RM_ cells (*18*), supporting their differentiation into T_RM_ cells. The magnitude of the CD8^+^ T cell response in the skin positively correlated with that present in the blood of the same patients, and both responses associated with protection from hospitalization in this cohort. These data provide insights into the biology and differentiation kinetics of T_RM_ cells in a human virus infection and support a protective role of skin-resident and circulating CD8^+^ T cells in dengue, with implications for vaccine design.

## RESULTS

### Activated and proliferating T cells are enriched in the skin in dengue

**Fig. 1.**
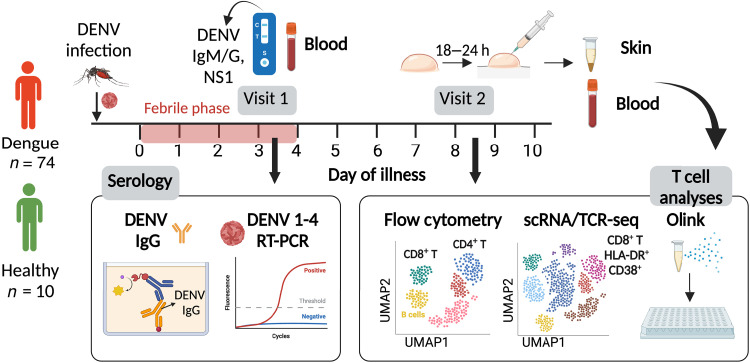
Study design and summary of investigations performed in this study. Day of illness is calculated from the day of fever onset. h, hours.

**Fig. 2.**
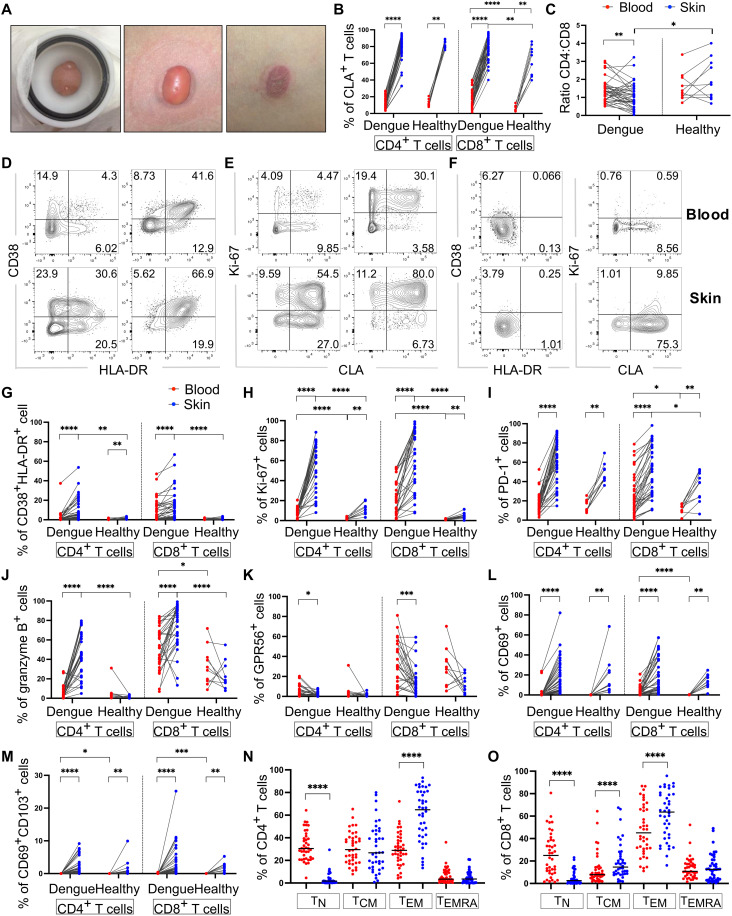
Activated and proliferating T cells are enriched in the skin of patients with dengue. Data from 98 matched blood and skin samples from *n* = 39 patients with dengue and *n* = 10 healthy volunteers. (**A**) Photograph of a representative skin blister induced on the forearm of a study participant using a clinical suction pump and chamber, 1 hour (left) and 2 hours (middle) from the start of the procedure, and after aspiration of fluid (right). (**B**) The percentages of blood and skin CLA^+^ CD4^+^ and CD8^+^ T cells are summarized for patients and healthy volunteers. (**C**) CD4^+^:CD8^+^ T cell ratios in the blood and skin of patients with dengue and healthy volunteers. (**D** to **F**) Representative flow cytometry plots of HLA-DR, CD38, and Ki-67 expression in blood and skin CD4^+^ and CD8^+^ T cells from a patient with dengue [(D) and (E)] and healthy volunteer [(F), shown for CD4^+^ T cells]. (**G** to **M**) Frequencies of HLA-DR^+^ CD38^+^ (G), Ki-67^+^ (H), PD-1^+^ (I) granzyme B^+^ (J), GPR56^+^ (K), CD69^+^ (L), and CD69^+^ CD103^+^ (M) CD4^+^ and CD8^+^ T cells expressing in skin and blood of patients with dengue and healthy volunteers. (**N** and **O**) Frequencies of blood and skin T cell subsets defined by CCR7 and CD45RA expression: T_N_ (naïve), CCR7^+^CD45RA^+^; T_CM_ (T central memory), CCR7^+^CD45RA^−^; T_EM_ (T effector memory), CCR7^−^CD45RA^−^; T_EMRA_ (T effector memory reexpressing CD45RA), CCR7^−^CD45RA^+^. Data points for blood and skin samples for each participant are shown in red and blue, respectively. Statistics were determined by Wilcoxon matched-pairs sign rank test between blood and skin and by Mann-Whitney *t* test between patients with dengue and healthy volunteers. For all figures: *P ≤ 0.05; **P ≤ 0.01; ***P ≤ 0.005; ****P ≤ 0.0001.

To investigate T cell responses in matched skin and blood compartments during DENV infection, we recruited 83 adult study participants, including 73 patients with confirmed dengue (40 ± 13 years, mean age ± SD) and 10 healthy volunteers with no serological evidence of prior DENV infection (42 ± 17 years, mean age ± SD; participant details in Table 1). Study participants donated blood and skin samples for research purposes at two time points (Fig. 1): Visit 1 time point (days 3 to 5 from fever onset) was chosen to differentiate primary from secondary DENV infection on the basis of DENV immunoglobulin G (IgG) titers, as described previously (*19*) and for identification of the DENV serotype of infection; visit 2 time point (days 7 to 10 from fever onset), which captured the peak of the T cell response in dengue (*20*, *21*), was chosen for blood and skin T cell analyses (Fig. 1). Skin T cells were obtained from skin suction blisters induced using a clinical grade pump as described (Fig. 2A) (*7*, *22*). On the following day, the fluid accumulated in the skin blister was collected and centrifuged to separate cell pellet from supernatant, both used for downstream analyses. DENV RNA could be detected in the supernatant of the skin suction blister (fig. S1), in line with previous findings in DENV-infected nonhuman primates, at a similar time postinfection (*23*). Analyses of the immune cells accumulating in the skin blister showed high expression of the skin-homing marker CLA compared with matched blood T cells, confirming that these cells are derived from the underlying skin tissue (Fig. 2B). These data confirmed that the used skin suction blister method allows isolation of T cells from the skin with minimal contamination of blood T cells.

**Table 1.** Clinical information for the patients with dengue and healthy volunteers included in the study. WHO, World Health Organization; N.A., not applicable; LOD, limit of detection.

	Patients with dengue (*n* = 74)	Healthy donors (*n* = 10)
**Age**		
Means ± SD	39 ± 13	42 ± 17
**Sex**		
Male	60 (81%)	7 (70%)
Female	14 (19%)	3 (30%)
**2009 WHO classification**		N.A.
Severe dengue	3 (4%)	
Dengue with warning signs	44 (59%)	
Dengue without warning signs	27 (37%)	
**Serostatus**		N.A.
Primary infection	40 (54%)	
Secondary infection	34 (46%)	
**Serotype**		N.A.
DENV1	2 (3%)	
DENV2	34 (46%)	
DENV3	19 (26%)	
DENV4	1 (1%)	
Unknown (<LOD)	18 (24%)	
**Hospitalization**		N.A.
Outpatient	21 (31%)	
Inpatient	31 (42%)	
Outpatient/inpatient*	20 (27%)	
**Thrombocytopenia**		N.A.
With	53 (72%)	
Without	21 (28%)	
**Rash**		N.A.
With	28 (38%)	
Without	46 (62%)	
**Highest hematocrit**		Not recorded
Means ± SD	45.7 ± 3.9	
**Lowest platelet count**		Not recorded
Means ± SD (×103/μl)	74.0 ± 51.3	

* Outpatient/inpatient refers to outpatients at visit 1 who were later admitted at visit 2.

We first investigated CD4^+^ and CD8^+^ T cell activation and proliferation within the skin and blood compartments of 49 participants (39 patients with dengue and 10 healthy volunteers) and asked whether skin T cells had distinct phenotypic and functional features compared with their circulating counterparts. Peripheral blood mononuclear cells (PBMCs) and cells from the skin blister aspirates, defined hereinafter as “blood” and “skin” cells, were analyzed by flow cytometry for expression of CD4^+^ and CD8^+^ T cell markers of activation and proliferation [Human Leukocyte Antigen - DR isotype (HLA-DR)], CD38, CD69, PD-1, and Ki-67), tissue residence (CD69, CD103, and CLA), differentiation (CCR7 and CD45RA), and cytotoxicity (granzyme B and GPR56). CD4^+^:CD8^+^ T cell ratios in the blood were similar in healthy volunteers and patients with dengue, while those in the skin of patients with dengue were lower compared with that of healthy volunteers, suggesting an expansion or influx of CD8^+^ T cells into the skin compartment during DENV infection (Fig. 2C). Furthermore, CD4^+^ and CD8^+^ T cells in the skin of patients with dengue displayed increased activation and proliferation compared with their blood counterparts (Fig. 2, D, E, and G to I). Low levels of activated and proliferating T cells could be detected in the skin, but not in the blood of healthy volunteers, suggesting that T cells in the skin may undergo turnover in steady state (Fig. 2, G to I). The frequencies of PD-1^+^ T cells were also significantly higher in the skin compared with the blood of healthy volunteers, in line with previous reports (Fig. 2I) (*24*). In patients with dengue, but not in healthy volunteers, skin CD4^+^ and CD8^+^ T cells contained higher frequencies of granzyme B^+^ cells compared with their blood counterparts (Fig. 2J). GPR56, a G protein–coupled receptor described to mark blood cytotoxic T cells (*25*, *26*), was expressed at higher levels in blood compared with skin CD4^+^ and CD8^+^ T cells of patients with dengue, while expression was similar across these two compartments in T cells from healthy volunteers (Fig. 2K). Skin CD4^+^ and CD8^+^ T cells from both patients with dengue and healthy volunteers expressed significantly higher levels of CD69 compared with their blood counterparts, with a proportion of skin CD69^+^ cells coexpressing CD103 (Fig. 2, L and M). CD69^+^ and CD69^+^ CD103^+^ CD8^+^ T cells were increased in the blood of patients with dengue compared with that of healthy volunteers, although their levels were lower compared with those of their skin counterparts (Fig. 2, L and M). In patients with dengue, skin CD4^+^ and CD8^+^ T cells were mainly CCR7^−^CD45RA^−^ [T effector memory (T_EM_) cells] followed by CCR7^+^CD45RA^−^ [T central memory (T_CM_)]. In contrast, blood contained CCR7^+^CD45RA^+^ [naïve (T_N_)], T_CM_, and T_EM_ CD4^+^ and CD8^+^ T cells and CD45RA^+^ [T effector memory RA reexpressing (T_EMRA_)] CD8^+^ T cells (Fig. 2, N and O). Similarly, CD4^+^ and CD8^+^ T cells in the skin of healthy volunteers comprised T_EM_ and T_CM_ cells, respectively, as well as T_EMRA_ cells for CD8^+^ T cells (fig. S2, A and B). In summary, our data demonstrate that activated and proliferating T cells expressing high levels of granzyme B were highly enriched in the skin compared with the blood of patients with dengue, suggesting that the skin is a key site for T cell immunosurveillance of DENV during acute infection.

### Marked reshaping of the skin T cell compartment in dengue

**Fig. 3.**
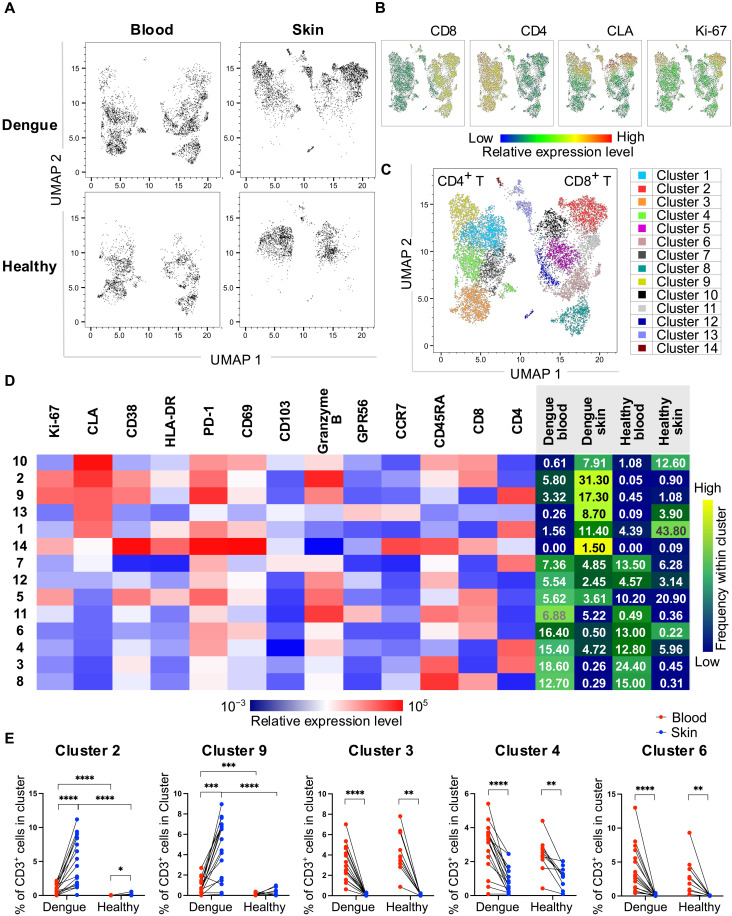
Skin and blood T cells are largely distinct. Data from 54 matched skin and blood samples from *n* = 17 patients with dengue and *n* = 10 healthy volunteers. (**A** and **B**) UMAP plots of blood and skin CD3^+^ T cells from patients with dengue and healthy volunteers. Expression of CD8, CD4, CLA, and Ki-67 within the UMAP is shown in (B). (**C**) The 14 T cell clusters identified by Phenograph within CD3^+^ cells are shown. Each color corresponds to a cluster. (**D**) Relative mean fluorescence intensities (MFIs) of the analyzed markers within each cluster are shown in the heatmap. The mean frequencies of T cells within each cluster in blood and skin of patients with dengue and healthy volunteers are shown in the columns (right; sum of the mean frequencies is 100% within each column). (**E**) Frequencies of cells within clusters enriched in the skin or blood of patients with dengue and healthy volunteers. Statistics were calculated by Wilcoxon matched-pairs sign rank test between blood and skin and by Mann-Whitney *t* test between healthy volunteers and patients.

To investigate the phenotypic and functional features of skin and blood T cells, we performed unsupervised analyses using dimensionality reduction algorithms. To this end, we selected flow cytometry standard files from 54 matched skin and blood samples derived from 17 patients with dengue and 10 healthy volunteers, which had been analyzed using identical flow cytometer settings. These files were downsampled to the same number of cells, concatenated and analyzed using uniform manifold approximation and projection (UMAP) and Phenograph cluster analysis to identify T cell populations enriched within blood or skin compartments. Our findings show that blood and skin CD4^+^ and CD8^+^ T cells mapped to distinct areas of the UMAP plots in both patients with dengue and healthy volunteers (Fig. 3A). A partial overlap of skin and blood CLA^+^ Ki-67^+^ T cells was observed in patients with dengue (Fig. 3, A and B), suggesting shared features between cells in these two compartments during infection. Phenograph analyses identified 14 different CD3^+^ T cell clusters, which included 5 CD4^+^ T cell and 7 CD8^+^ T cell clusters (respectively, clusters 1, 3, 4, 7, and 9 and clusters 2, 5, 6, 8, 10, 11, and 14; Fig. 3C and fig. S3) as well as 2 clusters with low CD4 and CD8 expression (clusters 12 and 13; Fig. 3C). The frequencies of T cells within these clusters and the relative mean fluorescence intensity (MFI) expression of the markers analyzed are summarized in a heatmap (Fig. 3D). These 14 clusters largely distinguished blood T cells from their skin counterparts, albeit with some clusters being shared between the two compartments. Skin T cells from healthy volunteers mapped predominantly within clusters 1 and 5 followed by cluster 10 and were largely nonoverlapping with their blood counterparts (Fig. 3D). In contrast, skin T cells from patients with dengue largely localized within clusters 2 and 9, which comprise CD8^+^ and CD4^+^ T cells, respectively, that are highly activated and proliferating, display cytotoxic capacity (granzyme B^high^) and high expression of CLA and CD69 but low expression of CD103 and CCR7 (Fig. 3D). These cells were enriched in the skin of patients with dengue, were present, albeit at lower frequencies, in their blood counterparts, and were largely absent in healthy volunteers (Fig. 3E). The phenotypic and functional features of these cells (CLA^+^, PD-1^+^, Ki-67^high^, CD69^+^, and granzyme B^high^) strongly resembled those of DENV-specific T cells detected in the blood of patients with dengue using DENV peptide-HLA class-I tetramers at days 7 to 10 of illness (*26*), which were shown to be distinct to that of “bystander activated” T cells in dengue that lack expression of CLA and are specific for antigens unrelated to DENV (*7*). On the basis of these considerations, these cells may represent bona fide DENV-specific T cells and are herein named “responding T cells.”

T cells in the blood of patients with dengue mapped predominately within clusters 3, 4, 6, and 8 followed by cluster 11 (Fig. 3D). The two most abundant clusters of CD4^+^ T cells (clusters 3 and 4) and CD8^+^ T cells (clusters 6 and 8) in the blood of patients with dengue displayed lower expression of HLA-DR, CD38, granzyme B, Ki-67, and CLA compared with T cells enriched in the skin. Cluster 11 containing PD-1^+^ CD45RA^+^ CCR7^−^ CD8^+^ T cells (T_EMRA_ cells) with high cytotoxic potential (granzyme B^+^ GPR56^+^) was present in the blood and skin of patients with dengue but largely absent in both compartments of healthy volunteers (Fig. 3D). In contrast, clusters 1 and 10 containing CD4^+^ and CD8^+^ T cells, respectively, expressing CLA, PD-1, and CD69 but lacking expression of activation and proliferating markers were present in the skin of both healthy volunteers and patients with dengue, although frequencies were higher in the former (Fig. 3D). These cells may be resting skin resident T cells. Overall, skin T cells in patients with dengue differed substantially from those in healthy volunteers, while blood T cells looked more similar between patients with dengue and healthy volunteers.

In summary, unsupervised analyses show that blood and skin T cells were largely phenotypically distinct in steady state and during DENV infection. In patients with dengue, skin T cells were markedly distinct from both their blood counterparts and skin T cells of healthy volunteers, suggesting that the skin compartment undergoes extensive reshaping during DENV infection.

### Skin and blood DENV-specific CD8^+^ T cells display similar features

**Fig. 4.**
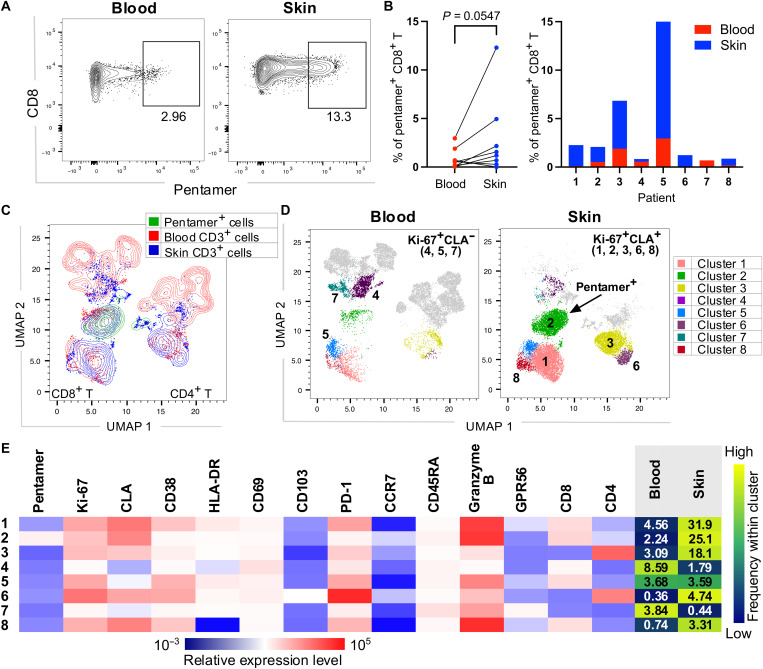
DENV pentamer^+^ and Ki-67^+^ CLA^+^ T cells are enriched in the skin. (**A**) Representative flow cytometry plots showing staining profiles for pooled DENV NS3_1608-1617_-HLA-A*11:01 and NS5_2610-2618_-HLA-A*11:01 pentamers (defined herein DENV pentamers) of CD3^+^ T cells in matched blood and skin of a patient with dengue. Pentamers were matched to the DENV serotype of infection (**B**) Frequencies of DENV pentamer^+^ CD8^+^ T cells in 16 matched blood and skin samples from *n* = 8 patients with dengue. (**C**) UMAP plots showing the localization of manually gated DENV pentamer^+^ CD8^+^ T cells (green) within blood (red) and skin (blue) CD3^+^ T cells in four matched blood and skin samples from *n* = 2 patients. (**D**) Shown are the clusters identified by Phenograph that correspond to Ki-67^+^CLA^+^ and Ki-67^+^CLA^−^ CD4^+^ and CD8^+^ T cells shown for blood and skin cells. (**E**) Heatmap showing MFI expression of the analyzed markers in Ki-67^+^CLA^+^ and Ki-67^+^CLA^−^ clusters from (D). The mean frequencies of each cluster are shown within blood and skin T cells. Statistics in (B) were calculated using Mann-Whitney *t* test.

As blood and skin T cells appeared to be largely distinct populations of cells, we next asked whether the features of DENV-specific CD8^+^ T cells are distinct in these two compartments. DENV-specific T cells were analyzed using peptide-HLA class I pentamers directly ex vivo. We previously showed that DENV NS3 and NS5 are the major targets of the CD8^+^ T cell response (*21*), with NS3_1608-1617_ and NS5_2610-2618_ epitopes being the most frequently recognized epitopes restricted to HLA-A*11:01, a commonly expressed HLA-type in the Singapore population (*26*, *27*). Here, we identified 8 HLA-A*1101^+^ patients with dengue within our cohort whose CD8^+^ T cells responded with detectable frequencies to at least one of these epitopes and analyzed their blood and skin cells by flow cytometry using HLA-A*11:01-NS3_1608-1617_ and NS5_2610-2618_ pentamers matched to the DENV serotype of infection for each patient. To evaluate the features of the DENV-specific T cells, cells were costained with HLA-A*11:01 pentamers and antibodies targeting phenotypic and functional T cell markers. The frequencies of CD8^+^ HLA-A*11:01-NS3_1608-1617_/NS5_2610-2618_ pentamer^+^ cells varied between patients but were consistently higher in the skin compared with the blood in six of eight patients, supporting our above findings of a more robust T cell response in the skin (Fig. 4, A and B). For two of these patients, we obtained a sufficient number of events to allow a more in-depth UMAP and Phenograph cluster analyses of pentamer^+^ skin versus blood CD8^+^ T cells. In both patients, the manually gated pentamer^+^ cells mapped to a single overlapping cluster of the UMAP and Phenograph plots (Fig. 4, C and D: cluster in green/cluster 2 and fig. S4). The proportions of pentamer^+^ skin CD8^+^ T cells were, on average, ~10-fold higher compared with those in the blood (Fig. 4E: cluster 2, 25.1% versus 2.24%; fig. S3). Cells in cluster 2 displayed a phenotype that is characteristic of DENV-specific CD8^+^ T cells, namely, CD45RA^+^ CCR7^low^ and with high expression of Ki-67, CLA, CD69, CD38, HLA-DR, PD-1, and granzyme B (Fig. 4E).

Our previous work using DENV peptide-HLA class-I tetramers showed that CLA and Ki-67 can be used to identify bona fide DENV-specific T cells that are responding to DENV (*26*). To analyze the broader pool of responding T cells, we therefore analyzed the features of Ki-67^+^ CLA^+^ CD4^+^ and Ki-67^+^ CLA^+^ CD8^+^ T cells (Fig. 4D). Ki-67^+^ CLA^+^ CD8^+^ T cells mapped to three distinct clusters: cluster 2 that contained the pentamer^+^ cells and clusters 1 and 8 that were adjacently located and contained CD8^+^ T cells expressing similar phenotypic and functional markers, except for HLA-DR that showed decreased expression in cluster 8 (Fig. 4, D and E). Ki-67^+^ CLA^+^ CD4^+^ T cells mapped to two adjacently located clusters (clusters 3 and 6). All five clusters of Ki-67^+^ CLA^+^ T cells were more abundant in the skin compared with matched blood Fig. 4E). We identified three clusters of cells that were Ki-67^+^ but did not express CLA; these were CD8^+^ T cells (clusters 4 and 5) or CD4^low^CD8^low^ T cells (cluster 7) and were enriched in blood compared with skin (clusters 4 and 7) or present at similar frequencies in these two compartments (cluster 5; Fig. 4, D and E).

In summary, we show that DENV-specific pentamer^+^ CD8^+^ T cells and Ki-67^+^ CLA^+^ CD4^+^/CD8^+^ T cells were highly enriched in the skin compared with blood. Skin and blood pentamer^+^ CD8^+^ T cells were located within the same cluster of cells, suggesting similar expression of the analyzed markers in DENV-specific skin and blood T cells and potentially reflecting recent migration of these cells from skin to blood.

### Increased CD8^+^ Ki-67^+^ CLA^+^ T cell responses in nonhospitalized patients with dengue

**Fig. 5.**
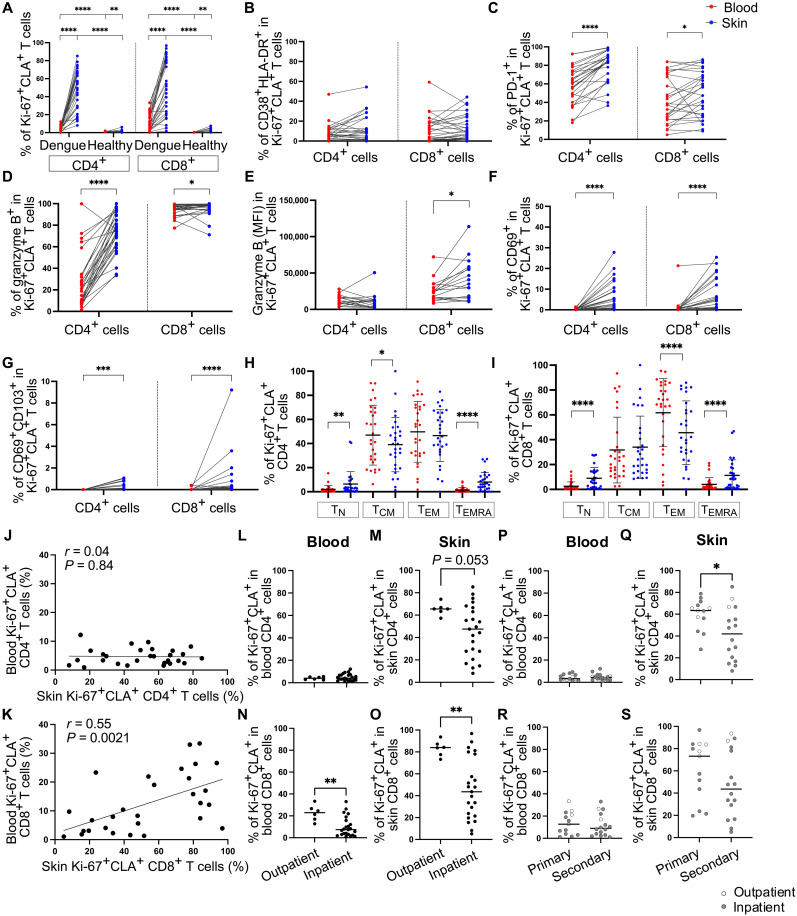
The frequencies of skin Ki-67^+^ CLA^+^ CD8^+^ T cells are directly correlated and associate with improved disease outcomes. Data from 78 matched skin and blood samples from *n* = 29 patients with dengue [(A) to (S)] and *n* = 10 healthy volunteers (A). (**A**) Frequencies of Ki-67^+^ CLA^+^ cells in blood and skin CD4^+^ and CD8^+^ T cells in patients with dengue and healthy donors. (**B** to **G**) Frequencies of Ki-67^+^ CLA^+^ CD4^+^ and Ki-67^+^ CLA^+^ CD8^+^ T cells expressing the indicated markers of activation proliferation, tissue residence, and cytotoxicity in the blood and skin. MFI values are also shown for granzyme B in (E). (**H** and **I**) Differentiation status of Ki-67^+^ CLA^+^ CD4^+^ and CD8^+^ T cells defined by expression of CCR7 and CD45RA. (**J** and **K**) Correlation of blood and skin Ki-67^+^ CLA^+^ CD4^+^ (J) and Ki-67^+^ CLA^+^ CD8^+^ T cells (K) using Spearman’s rank correlation test. (**L** to **O**). Frequencies of Ki-67^+^ CLA^+^ CD4^+^ [(L) and (M)] and CD8^+^ T cells [(N) and (O)] are shown in patients stratified by patient hospitalization status. (**P** to **S**) Frequencies of Ki-67^+^ CLA^+^ CD4^+^ [(P) and (Q)] and CD8^+^ T cells [(R) and (S)] are shown in patients stratified by primary/secondary infection. Statistics were calculated using Wilcoxon matched-pairs sign rank test between blood and skin and using Mann-Whitney *t* test between healthy volunteers and patients and between inpatients and outpatients.

We further investigated the relationship between skin and blood responding Ki-67^+^ CLA^+^ T cells in a larger number of patient samples and asked whether the frequencies of these cells in skin and/or blood correlate with dengue disease outcomes. To achieve this, we compared the frequencies and phenotypic/functional features of Ki-67^+^ CLA^+^ CD4^+^ and Ki-67^+^ CLA^+^ CD8^+^ T cells in 78 matched skin and blood samples from 29 patients with dengue and 10 healthy volunteers. The frequencies of Ki-67^+^ CLA^+^ T cells were notably higher in patients with dengue compared with healthy volunteers and in skin compared with blood (Fig. 5A). In patients with dengue, Ki-67^+^CLA^+^ T cells in skin and blood showed similar coexpression of HLA-DR and CD38 (Fig. 5B). However, the frequencies of cells expressing PD-1, granzyme B, and the skin residency markers CD69/CD103 were significantly higher in skin than in blood Ki-67^+^ CLA^+^ CD4^+^ and CD8^+^ T cells (Fig. 5, B to G). Skin and blood Ki-67^+^ CLA^+^ CD4^+^ and CD8^+^ T cells were predominantly T_CM_ and T_EM_ cells, although skin Ki-67^+^ CLA^+^ T cells contained a minor fraction of cells that were phenotypically naïve and T_EMRA_ cells (Fig. 5, H and I). The frequencies of skin Ki-67^+^ CLA^+^ CD4^+^ T cells varied largely amongst patients and did not correlate with those present in matched blood, suggesting that generation of these cells may be independently controlled (Fig. 5J). In contrast, the frequencies of Ki-67^+^ CLA^+^ CD8^+^ T in the skin directly correlated with those present in the blood, suggesting that there may be recirculation of these cells between the two compartments (Fig. 5K). To assess the correlation of T cell responses with clinical outcomes, we stratified patients on the basis of whether they required hospitalization (inpatients) or were deemed fit to return to their home (outpatients), following a clinical assessment by expert clinicians. Hospitalization status correlated with parameters of disease severity, specifically with having dengue warning signs, thrombocytopenia, and low platelet count (table S1). This is consistent with the criteria of hospitalization for patients with dengue in Singapore, which is based on clinical and laboratory parameters (*28*).

The frequencies of skin Ki-67^+^ CLA^+^ CD4^+^ T cells were highest in patients with primary infection and showed a higher trend in outpatients compared with inpatients, while no differences were observed for blood Ki-67^+^ CLA^+^ CD4^+^ T cells (Fig. 5, L and M and P and Q). In contrast, outpatients displayed higher frequencies of both blood and skin Ki-67^+^ CLA^+^ CD8^+^ T cells compared with inpatients. Patients with primary DENV infection displayed increased trends of Ki-67^+^ CLA^+^ CD8^+^ T cells, although these differences were not statistically significant (Fig. 5, N, O, R, and S).

In summary, we show that responding Ki-67^+^ CLA^+^ CD4^+^ and CD8^+^ T cells were enriched in the skin compartment. The frequencies of Ki-67^+^ CLA^+^ CD8^+^ T cells in skin and blood were directly correlated and were increased in patients who did not require hospitalization compared with those that did, suggesting a protective role of CD8^+^ T cells.

### Increased T cell cytokine responses in nonhospitalized patients

**Fig. 6.**
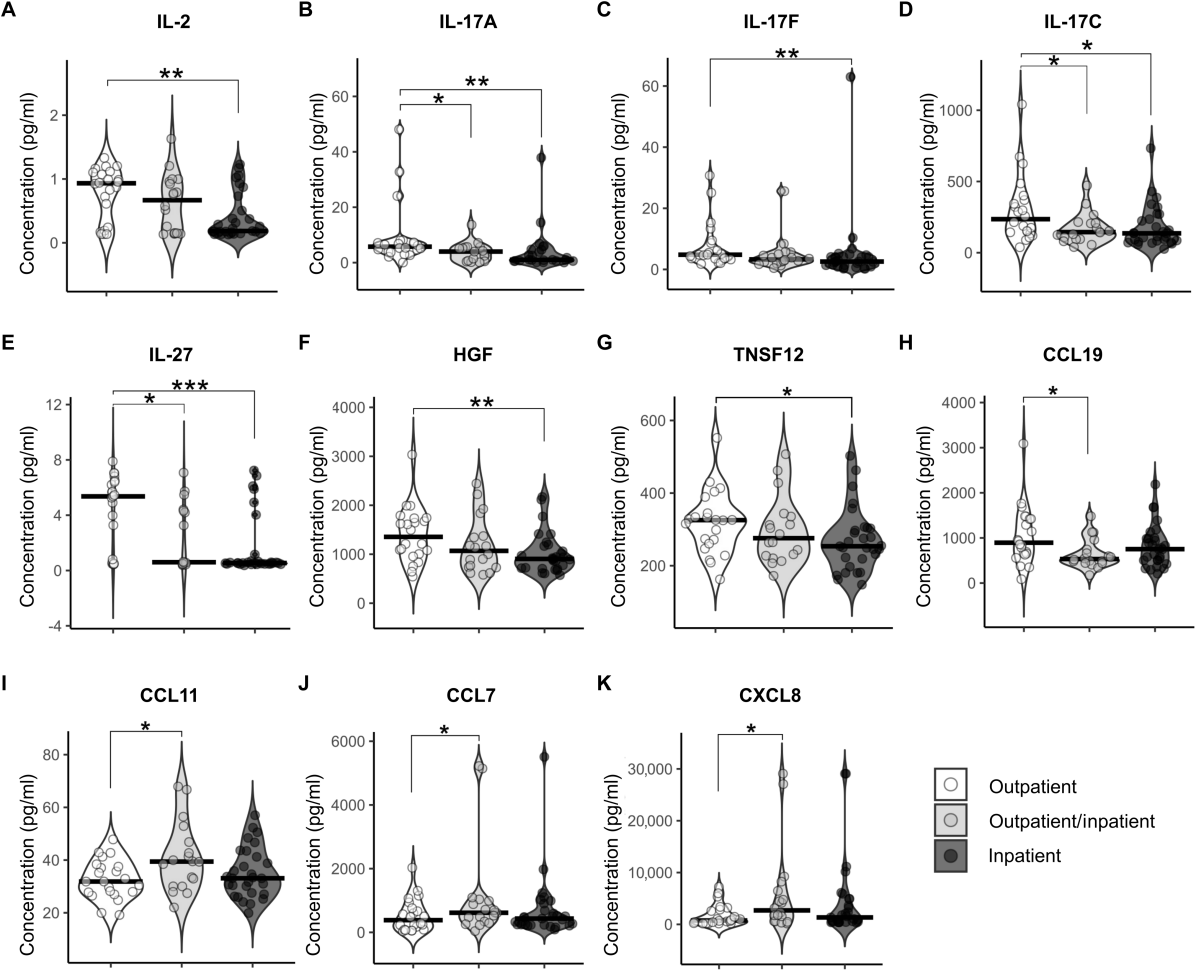
Increased T cell–related cytokines in the skin correlate with improved clinical outcomes. Cytokines were measured using Olink Target 48 proteomics in skin blister fluids of *n* = 69 patients with dengue. Shown are the concentrations (picograms per milliliter) of IL-2 (**A**), IL-17A (**B**), IL-17F (**C**), IL-17C (**D**), IL-27 (**E**), HGF (**F**), TNSF12 (**G**), CCL19 (**H**), CCL11 (**I**), CCL7 (**J**), and CXCL8 (**K**). Data are stratified by patients’ hospitalization status (outpatient, *n* = 22; outpatient/inpatient, *n* = 18; inpatient, *n* = 29). Statistics were calculated by one-way analysis of variance (ANOVA).

To confirm whether there was a superior skin T cell response in outpatients compared with inpatients on a larger number of patient samples, we analyzed skin blister fluid supernatants from 69 patients, including 22 outpatients, 18 outpatients/inpatients, and 29 inpatients, for the presence of 45 cytokines/chemokines using the Olink Target 48 Cytokine panel. Outpatients/inpatients were outpatients at visit 1 but were later hospitalized because of worsening conditions. Outpatients displayed higher levels of interleukin-2 (IL-2), IL-17A, IL-17F, and IL-17C in the skin compared with inpatients, with a stepwise decrease of these cytokines from outpatients to outpatients/inpatients and inpatients (Fig. 6, A to D). IL-2 and IL-17 cytokines are part of the core transcriptional signature described for CD4^+^ and CD8^+^ T_RM_ cells (*18*). The levels of IL-27, a member of the IL-12 cytokine family, which supports T cell differentiation, were also higher in outpatients compared with outpatients/inpatients and inpatients (Fig. 6E). Outpatients also displayed higher levels of hepatocyte growth factor (HGF), tumor necrosis factor superfamily member 12 (TNFSF12), and CCL-19, a chemokine that mediates recruitment of CCR7^+^ T cells (Fig. 6, F to H). In contrast, chemokines associated with the recruitment of cell-types including leukocytes, neutrophils, and eosinophils (respectively, CCL7, CXCL8, and CCL11) were increased in outpatients/inpatients compared with outpatients, although differences were less notable (Fig. 6, I to K). In summary, our data show increased levels of T cell–related cytokines including IL-2, IL-17, and IL-27 in the skin of outpatients compared with that of inpatients, supporting the association of a superior skin T cell response with improved clinical outcomes in this dengue cohort.

### Activated skin CD8^+^ T cells display features of T_RM_ cells

**Fig. 7.**
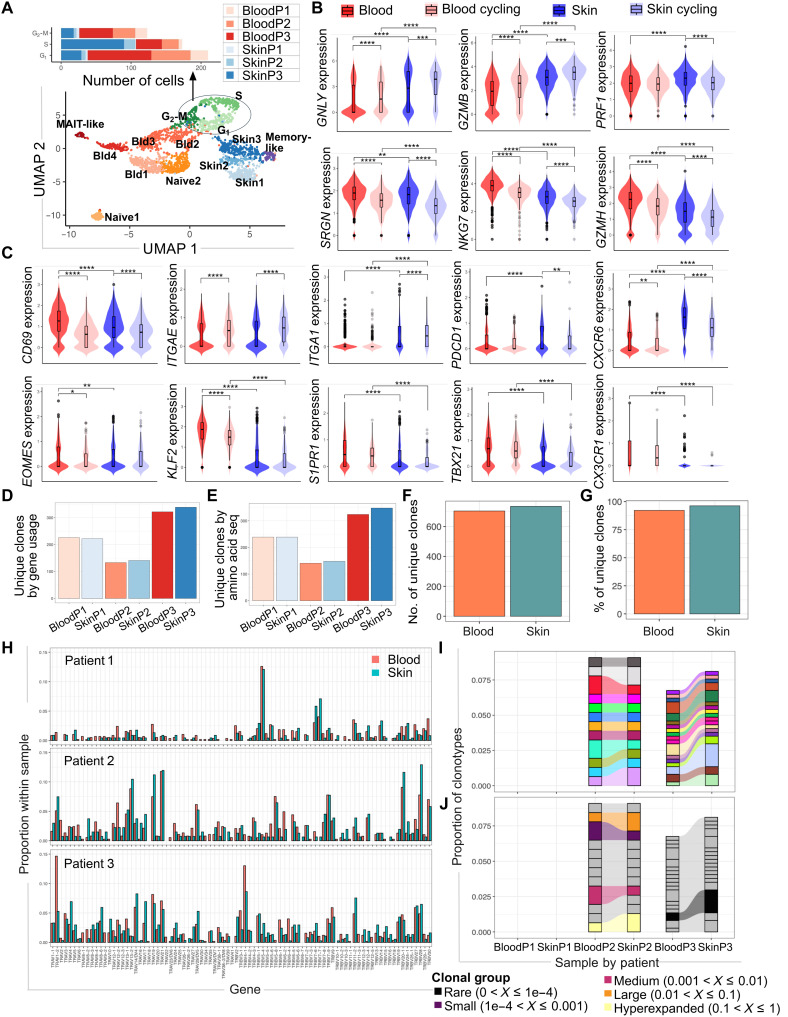
Activated CD8^+^ T cells in the skin in dengue display features of T_RM_ cells. (**A**) UMAP plot showing the distribution of skin (blue), blood (red-orange), and cycling (green) CD8^+^ T cells based on single-cell gene expression. The number of cells from blood and skin of each patient, which are located in the different phases of the cell cycle, is indicated in the top graph. (**B**) Gene expression levels of genes involved in cytotoxicity are shown for CD8^+^ T cells and cycling CD8^+^ T cells in blood and skin. *GNLY*, granulysin; *GZMB*, granzyme B; *PRF1*, perforin; *SRGN*, serglycin; *NKG7*, natural killer cell granule protein 7; *GZMH*, granzyme H. (**C**) Expression levels are shown for genes known to be up-regulated or down-regulated in T_RM_ cells (top and bottom, respectively) in blood and skin CD8^+^ T cells and cycling CD8^+^ T cells. (**D** to **H**) Number of unique clones defined by TRAV and TRBV gene usage is shown for blood and skin CD8^+^ T cells in each patient (D) and summarized for all patients (G). Expression of TRAV and TRVB genes in blood and skin activated CD8^+^ T cells is shown for each patient (E). The number of unique clonotyes defined by CDR3 regions is shown for each patient (F), and the percentage of unique clonotypes is summarized for all patients (H), downsampled by each patient’s lower limit of cell counts. (**I**) The proportion of clonotypes, defined by the CDR3 amino acid sequences, shared by skin and blood samples is shown for each patient after downsampling for each patient’s lowest cell count sample. Colors identify unique clonotypes. (**J**) Shared clonotypes that are expanded (proportion > 0.01) in skin and blood of patients 2 and 3 are shown. Statistics in (B) and (C) are calculated using one-way ANOVA with Benjamini-Hochberg correction. P1, P2, and P3 indicates patients 1, 2, and 3, respectively.

To gain a more in-depth understanding of the features and clonal relationship of skin and blood T cell responses in dengue, we performed paired gene expression and T cell receptor (TCR) analyses by 10x Genomics single-cell RNA sequencing (scRNA-seq) and TCR sequencing (TCR-seq) of matched skin and blood activated (HLA-DR^+^ CD38^+^) CD8^+^ T cells isolated by cell sorting (days 7 to 10 from fever onset; patient details in table S2 and Table 1). scRNA/scTCR-seq was performed on a combined total of 8175 single cells derived from six matched skin and blood samples of three patients with dengue. UMAP analyses of the gene expression data identified clusters of T cells that were clearly distinct between blood and skin and a cluster of proliferating cells (cycling) that contained both skin and blood CD8^+^ T cells (Fig. 7A). Skin CD8^+^ T cells displayed increased expression of genes encoding for granzyme B (*GZMB*), granulysin (*GNLY*), and perforin (*PRF1*), which are critical for cellular cytotoxicity, with granulysin and granzyme B expression progressively increasing in clusters corresponding to blood, blood cycling, skin, and skin cycling CD8^+^ T cells. However, other genes also linked to cytotoxicity displayed increased expression in blood CD8^+^ T cells compared with their skin counterparts [serglycin (*SRGN*), natural killer cell granule protein 7 (*NKG7*), and granzyme H (*GZMH*)] (Fig. 7B). Skin CD8^+^ T cells displayed increased expression of the skin and peripheral tissue residency markers CD69, CD103 (*ITGAE*), CD49 (*ITGA1*), CXCR6, and PD-1 (*PDCD1*), compared with their blood counterparts. Conversely, *EOMES*, *KLF2*, and *TBX21*, which encode for transcription factors described to be down-regulated in T_RM_ cells [respectively, Eomes, Krüppel factor 2 (KLF2), and T-bet] and S1PR1 (sphingosine-1-phosphate receptor 1), displayed decreased expression in skin compared with blood CD8^+^ T cells. CX3CR1 expression was also decreased in skin T cells compared with their blood counterparts (Fig. 7C). These data suggest that CD8^+^ T cells activated during DENV infection differentiate into T_RM_ cells by days 7 to 10 from illness onset.

We next measured the clonal diversity of matched skin and blood CD8^+^ T cells and their clonotype overlap. To this end, we analyzed the expression of TCRα and TCRβ chain variable genes (*TRAV* and *TRBV*) and the unique complementarity-determining regions (CDR3) that determine the CD8^+^ T cell clonotypes. For all patients analyzed, there was extensive sharing of *TRAV* and *TRBV* gene usage between skin and blood CD8^+^ T cells (Fig. 7, D and E). Analyses of the unique CDR3-defined clonotypes showed similar number of clonotypes in matched blood and skin CD8^+^ T cells, with a total of >600 unique clonotypes identified across the three patients, comprising >80% of clonotypes within the activated CD8^+^ T cell population (Fig. 7, F to H). Analyses of the proportion of clonotype sharing by skin and blood CD8^+^ T cells revealed 12 and 17 shared clonotypes in patients 2 and 3, respectively (Fig. 7I), with 4 of these clonotypes being expanded in size, ranging from small to large/hyperexpanded clonal groups (clonotype proportions of ≤0.01 and >0.01, respectively; Fig. 7J). These shared CD8^+^ T cell clonotypes accounted for ~7 to 9% of total clonotypes within activated CD8^+^ T cells of patients 2 and 3. We did not identify shared clonotypes between skin and blood CD8^+^ T cells of patient 1 (Fig. 7I).

In summary, these data show that activated and responding skin CD8^+^ T cells in DENV infection displayed transcriptional features of T_RM_ cells, supporting the differentiation of these cells into long-lived memory T cells within the skin tissue at the time of infection. Tissue-resident and circulating CD8^+^ T cells exhibited a moderate clonotypic diversity and some evidence of clonotype sharing in two of the three patients, suggesting either a common T cell precursor giving rise to circulating and skin T_RM_ cell clonotypes or recirculation of T_RM_ cells between tissues and blood during infection. Further studies are needed to define the extent of trafficking of T cells responding to DENV infection between the blood and skin compartments and potential links with disease outcomes.

## DISCUSSION

In this study, we show that CD4^+^ and CD8^+^ T cell responses in DENV infection were highly enriched in the skin where these cells displayed phenotypic and functional features of T_RM_ cells. Responding CD4^+^ and CD8^+^ T cells in the skin displayed increased expression of skin residency markers CD69 and CD103 and of PD-1 and granzyme B, compared with their blood counterparts. scRNA-seq of activated CD8^+^ T cells in the skin and blood showed distinct gene expression profiles of these cells. Skin CD8^+^ T cells expressed transcriptional signatures of human T_RM_ cells (*18*), supporting their differentiation into T_RM_ cells during acute infection. The magnitude of CD8^+^ T cell responses in the skin mirrored that of their blood counterparts, while skin CD4^+^ T cell frequencies were independent from those of matched blood CD4^+^ T cells, suggesting a differential regulation of CD4^+^ and CD8^+^ T cell responses between these compartments. Outpatients, who did not require hospitalization, displayed a higher magnitude of skin and blood CD8^+^ T cell responses and increased expression of skin T cell–related cytokines compared with inpatients. This study provides insights into the biology and dynamics of T_RM_ cells in a human acute viral infection and suggests a protective role of both skin and blood CD8^+^ T cells in dengue.

We previously showed in patients with dengue that circulating DENV-specific T cells express high levels of CLA while human cytomegalovirus (CMV)–specific T cells that are bystander activated during DENV infection lack expression of CLA (*7*, *26*). These studies showed that coexpression of Ki-67 and CLA distinguishes circulating DENV-specific T cells from bystander activated T cells. From these studies, it remained unclear whether Ki-67^+^ CLA^+^ DENV-specific T cells differentiate into T_RM_ cells in the skin and whether T cells responding in the skin and blood are phenotypically and functionally distinct.

Here, we show that CD4^+^ and CD8^+^ Ki-67^+^ CLA^+^ T cells are highly enriched in the skin compared with the blood compartment, suggesting their preferential localization within this site.

At days 7 to 10 from illness onset (corresponding approximately to days 11 to 14 from infection), responding CD8^+^ T cells in the skin displayed transcriptional and phenotypic features of T_RM_ cells. These cells expressed a core T_RM_ gene signature that includes up-regulation of CD69 and down-regulation of S1PR1 and KLF2 that collectively lead to T cell retention within tissues, up-regulation of adhesion molecules (CD103 and CD49a) and PD-1, modulation of chemokine receptor expression (CXCR6 and CX3CR1), and down-regulation of transcriptional factors Eomes and T-bet (*18*). Skin CD8^+^ T cells displayed increased expression of genes encoding for granzyme B, perforin, and granulysin. Consistently, flow cytometry analyses showed notably higher frequencies of granzyme B^+^ CD4^+^ and CD8^+^ T cells in the skin compared with those in the blood of the same patients, supporting a strong cytotoxic capacity of skin T cells in DENV infection. These data are consistent with previous studies in healthy individuals showing that, despite the low expression of perforin and granzymes by skin CD8^+^ T_RM_ cells in steady state, these cells can efficiently induce expression of these molecules upon cytokine or TCR stimulation (*29*).

Of note, the site of induction of the skin blister is unlikely to be the site of the DENV infection; our data therefore suggests that T cells responding to DENV will disseminate systemically across the skin tissue. These findings are in line with data in mouse models showing systemic migration of T_RM_ cells within the skin tissue during viral infection (*9*).

Previous studies in mice showed that virus-specific CD8^+^ T cells are highly polyclonal with >1000 different clonotypes responding to a single immunodominant virus epitope (*30*). This polyclonal response, which comprises T cells with differing levels of TCR avidity, ensures protection toward potential pathogen escape mutants (*31*). Similarly, in humans, CD8^+^ T cells targeting the immunodominant CMV NLV epitope display high clonality, with >5000 different clonotypes targeting the same epitope (*32*). Here, we show that the clonotype diversity of activated CD8^+^ T cells in DENV infection was low/moderate and similar in skin and blood compartments, with ~150 to 350 unique clonotypes identified per patient sample. Patient 2 displayed the lowest clonotype diversity and was experiencing a secondary DENV infection, while patients 1 and 3 were primary dengue cases. While a lower clonotype diversity in secondary DENV infection is consistent with a preferential reactivation of memory T cells generated in the primary infection, further studies on a higher number of patients are needed to study clonotypic diversity in primary and secondary DENV infection and address potential links with disease outcomes. In patients 2 and 3, we detected some clonotype sharing between skin and blood (~7%). Increasing evidence in mouse models suggests that some circulating T cells are already preconditioned to preferentially give rise to the T_RM_ cell lineage before entering the tissue (*33*, *34*). This contrasts earlier work suggesting that T_RM_ cell lineage decisions are made within tissues or the local draining lymph nodes following cues from dendritic cells and the local environment (*35*). Our findings cannot distinguish between these two possibilities as the observed clonotype sharing between circulating and skin T cells may be due to either a shared common precursor giving rise to cells in both compartments or the recirculation of T cells within the two compartments. Further studies are needed to understand the mechanisms driving T_RM_ cell lineage commitments in humans.

A limitation of our analyses is the small number of patient samples included in the scRNA-seq and the peptide-HLA pentamer flow cytometry analyses. The latter was due to difficulties in identifying patients with dengue expressing HLA-A*11:01 (~30% of patients in this cohort) and HLA-A*11:01^+^ patients who displayed a detectable response to the two epitopes analyzed (NS3_1608-1617_ and NS5_2610-2618_), despite these being the most immunodominant epitopes restricted to this HLA type (*26*, *27*). This study was not powered to compare T cell responses in patients infected with different DENV serotypes. The predominant serotype of infection in this cohort was DENV 2 (47%) followed by DENV 3 (25%) with DENV 1 and DENV 4 infections being less common (4%). For 25% of patients, we were unable to determine the serotype of infection as their RNAemia was below the detection limit at enrolment.

While most of the T cell response in dengue comprised Ki-67^+^CLA^+^ cells, we detected three distinct clusters of Ki-67^+^ CLA^−^ CD8^+^ T cells, two of which were highly enriched in the blood compared with the skin compartment, while a small cluster of cells (~3% of CD8^+^ T cells) was equally represented in the two compartments. Ki-67^+^ CLA^−^ CD8^+^ T cells may represent bystander activated CD8^+^ T cells that were primed in sites different from the skin or migrated from the skin to the blood and lost CLA expression. Previous studies showed that virus infection may trigger activation of T cells specific for persistent herpesviruses (human CMV and Epstein-Barr virus), and this is likely mediated by IL-15 produced during the infection (*7*, *36*). In this study, due to limiting number of cells present in the skin blisters, we were unable to address the antigen-specificity of these cells and the signals driving their activation.

Our study shows that Ki-67^+^ CLA^+^ CD8^+^ T cells, which phenotypically and functionally mirror DENV-specific CD8^+^ T cells identified using peptide-HLA tetramers (*26*, *27*, *37*), were present at higher frequencies in the skin and blood of patients with milder disease, who did not require hospitalization. Consistently, cytokines produced by effector T cells (IL-2 and IL-17) or cytokines/chemokines that drive differentiation and recruitment of these cells were increased in skin blisters from outpatients compared with those from inpatients. IL-17 is expressed by human CD4^+^ T_RM_ and CD8^+^ T_RM_ cells and was also shown to be induced in T cells of healthy volunteers by mosquito saliva in a human challenge study (*38*).

In summary, this study shows that a large proportion of the T cell response to DENV resides within the skin and suggests a protective role of T_RM_ and circulating CD8^+^ T cells. Our findings warrant evaluation of vaccine strategies that elicit priming of CD8^+^ T_RM_ cells.

## MATERIALS AND METHODS

### Study design and study participants

Patients with dengue (*n* = 74) and healthy volunteers (*n* = 10) included in this study were recruited at Tan Tock Seng Hospital and National Centre for Infectious Diseases between June 2019 and April 2023, Singapore, after written informed consent following Institutional Review Board approval (National Healthcare Group Domain Specific Review Board reference: 2018/00874). Clinical and demographic details of patients and healthy volunteers are summarized in Table 1. Dengue disease classification was performed by trained clinicians at the Tan Tock Seng Hospital/National Centre for Infectious Diseases and was based on the World Health Organization 2009 definition (*39*).

Inclusion criteria for patients with dengue include a confirmed diagnosis of dengue fever (NS1 positive and/or serology positive), no other diagnosis supporting the febrile episode, and ≥21 years of age. Inclusion criteria for healthy volunteers include not being diagnosed with dengue and with no serological evidence of past DENV infection and ≥21 years of age. Exclusion criteria for patients with dengue and healthy volunteers include pregnancy and breastfeeding (for women), having asthma, and existing skin conditions or being immunocompromised. Whole blood was collected at visits 1 and 2 (days 3 to 5 and days 7 to 10 from fever onset) in K3EDTA tubes, and skin suction blisters were raised at visit 2 in the same volunteers.

### DENV IgG

The Panbio Dengue IgG Indirect ELISA test was performed using plasma samples from patients with dengue and healthy individuals according to the manufacturer’s protocol (Abbott, catalog no. 01PE30). The absorbance was read using the Varioskan LUX multimode microplate reader. Primary and secondary DENV infection were defined on the basis of serum DENV IgG levels at days 3 to 5, as defined previously (*19*).

### Viral RNA extraction and DENV serotype analyses

The extraction of viral RNA from plasma was carried using the QIAamp Viral RNA Mini Kit (QIAGEN), following the manufacturer’s protocol. DENV serotype of infection was determined using the Center for Disease Control and Prevention DENV-1-4 real-time RT-PCR (qRT-PCR) Assay protocol with the Invitrogen SuperScript III One-Step Quantitative RT-PCR kit (Invitrogen, catalog no. 11732-088) on the Roche LightCycler 96. The primers and probes used are listed in table S3.

### DNA extraction and HLA-A typing

The DNA extraction protocol was adapted and modified from the QIAGEN AllPrep DNA/RNA Mini Kit. Briefly, 0.5 ml of clotted blood was mixed with RLT buffer and β-mercaptoethanol. The solution was vortexed and then incubated at room temperature for 5 min. The blood-RLT buffer solution (600 μl) was transferred to an AllPrep DNA spin column and spun down at 10,000 rpm for 1 min at 18°C. The flowthrough was discarded, and the process repeated until all the blood-RLT buffer solution had been spun down. The spin column was then washed twice with 500 μl of Buffer AW1 at 10,000 rpm for 1 min at 18°C, discarding the flowthrough for each wash. The spin column was washed once with 500 μl of Buffer AW2 and spun down at 14,000 rpm for 2 min at 18°C, discarding the flowthrough. The spin column was spun down once more at 14,000 rpm to dry the column. Buffer EB (50 μl), preheated to 70°C, was added to the spin column and let sit for 2 min before it was centrifuged at 10,000 rpm for 1 min. The process was repeated once more without discarding the flowthrough until the final volume of DNA eluted was ~100 μl. The DNA concentration was measured using a NanoDrop 2000. The HLA-A typing assay was adapted and modified from a previous study (*40*). For every reaction, a mix of 0.5 μl of deoxynucleotide triphosphates (dNTP) mix (Promega, catalog no. U1511), 2.5 μl of (NH_4_)SO_4_-Cl_2_ buffer, 2 μl of MgCl_2_, 0.25 μl of *Taq* DNA polymerase (Thermo Fisher Scientific, catalog no. EP0402), and 2 μl of each primer (A1101-AL#6 sequence: CGG AAT GTG AAG GCC CAG and A1101-AL#1 sequence: TCT CTG CTG CTC CGC CG) was prepared to a total volume of 9.25 μl. The DNA sample of interest or positive control (100 ng) was added to the mix, and nuclease-free water was topped up to a total volume of 25 μl per polymerase chain reaction (PCR) reaction. As a negative control, nuclease-free water was added in place of DNA. Thermocycling parameters were as follows: stage 1, 1 cycle of 96°C for 15 min; stage 2, 5 cycles of 96°C for 1 min, 66°C for 1 min, and 72°C for 1 min; stage 3, 40 cycles of 96°C for 1 min, 56°C for 1 min, and 72°C for 1 min; and final hold at 4°C. PCR products were analyzed by agarose gel electrophoresis.

### Blood processing and PBMC isolation

Whole blood was collected in K3EDTA tubes. The whole blood (1 ml) was aspirated into a 1.5-ml Eppendorf tube. The tube is spun down in a benchtop centrifuge at 3000 rpm for 5 min. The clotted blood was used for DNA extraction for HLA typing. PBMCs were isolated from the remaining whole blood by Ficoll-Paque gradient centrifugation, as previously described (*41*). Freshly isolated PBMCs were used for flow cytometry and RNA sequencing assays on the day of collection.

### Skin blister induction

The skin blister suction method was adapted from previous studies (*7*, *22*). Using a clinical pump connected to a chamber, blisters were induced on the forearm of the study volunteers. A negative pressure of 25 to 40 kPa (200 to 300 mmHg) below atmospheric pressure was applied for 2 to 4 hours until a blister was formed. After 18 to 24 hours, the blister fluid was aspirated using a sterile 23-gauge needle and a 2-ml syringe and transferred to a 1.5-ml Eppendorf tube. Skin cells were recovered by centrifugation at 3000 rpm for 10 min at 4°C. The blister fluid supernatant was cryopreserved and used for Olink Target 48 assay. The skin cell pellet was resuspended in 200 μl of AIM-V (Gibco, catalog no. 12055091) and 2% human serum medium for analysis. Freshly isolated cells from the skin blister were used for flow cytometry and RNA sequencing assays on the day of collection.

### Flow cytometry

Staining was performed on freshly isolated matched PBMCs and skin cells on the day of collection. Cells were stained with LIVE/DEAD Fixable Blue Stain for 10 min in the dark at room temperature. Cells were then stained with antibodies targeting surface markers in staining buffer (phosphate-buffered saline and 1% bovine serum albumin) and with phycoerythrin-labeled DENV pentamers for 20 min on ice. For panels with Ki-67 and granzyme B staining, cells were suspended with 100 μl of eBioscience FoxP3 Fixation/Permeabilization solution (Invitrogen, catalog no. 00-5523-00) for 45 min on ice before washing with Permeabilization buffer (Invitrogen, catalog no. 00-5523-00). Cells were then stained with antibodies targeting intracellular markers for 30 min on ice before acquisition on the BD LSRFortessa. The list of antibodies and peptide-HLA pentamers used is provided in tables S4 and S5, respectively. Flow cytometry data were analyzed on FlowJo Version 10; plugins include DownSample, UMAP, PhenoGraph, and ClusterExplorer.

### Olink Target 48 analysis

Analyses of the following 45 cytokines/chemokines were performed in skin blister fluid supernatant according to the manufacturer’s instructions using the Olink Target 48 Cytokine panel: IL-18, HGF, chemokine (C-C motif) ligand 19 (CCL19), CCL2, matrix metalloproteinase 12 (MMP12), lymphotoxin-alpha (LTA), Fms-related tyrosine kinase 3 ligand (FLT3LG), tumor necrosis factor, IL-17A, IL-2, IL-17F, colony-stimulating factor 3 (CSF3), IL-1β, oxidized low-density lipoprotein receptor 1 (OLR1), TNFSF12, C-X-C motif chemokine ligand 10 (CXCL10), vascular endothelial growth factor A (VEGFA), IL-33, thymic stromal lymphopoietin (TSLP), interferon-γ, CCL4, TGFA, IL-13, CXCL8, CCL8, IL-6, CCL13, CSF2, CCL7, IL-4, TNFSF10, oncostatin-M (OSM), MMP1, epidermal growth factor, IL-7, IL-15, CSF1, CXCL9, CXCL11, IL-17C, CXCL12, CCL11, IL-10, CCL3, Epstein-Barr virus-induced gene 3 (EBI3), and IL-27. Forty-five oligonucleotide conjugated antibody pairs were hybridized with skin blister fluid and incubated overnight at 4°C. Oligonucleotides brought into proximity hybridize, with extension facilitated by a DNA polymerase that results in the formation of a target-specific double stranded DNA barcode. The 45 unique DNA barcode sequences were then amplified by PCR before being quantified using the Olink Signature Q100.

### Single-cell RNA sequencing

CD8^+^ CD38^+^ HLA-DR^+^ cells from matched PBMCs and skin blister cells of patients with dengue were sorted into AIM-V and 2% human serum on the BD FACSAria III Sorter following staining with antibodies targeting surface markers. scRNA-seq of the sorted cells was carried out according to the 10x Genomics’ Chromium Next GEM Single Cell 5′ Reagent Kits v2 protocol. Briefly, sorted cells were mixed with 10x barcoded gel beads and loaded onto Chromium Next GEM Chip K, after which the assembled chip was run on the Chromium Controller X to generate GEMs (1000263 Chromium Next GEM Single Cell 5′ Kit v2, 1000286 Chromium Next GEM Chip K Single Cell Kit, and 1000190 Library Construction Kit, 10x Genomics). Post–GEM-RT cleanup was then carried out, followed by cDNA amplification according to the manufacturer’s protocol. Subsequently, V(D)J amplification from cDNA, V(D)J library construction, and 5′ gene expression library construction were carried out according to the manufacturer’s protocol (1000252 Chromium Single Cell Human TCR Amplification Kit, 10x Genomics). Quality control was performed on the Agilent 2100 Bioanalyzer G2938C. Libraries were sequenced paired-end 150 base pairs on the Illumina HiSeq X System (20,000 read pairs per cell).

### scRNA-seq data processing and analysis

Raw sequencing data were processed using the Cell Ranger multi pipeline (v7.1.0, 10x Genomics). The filtered gene-barcode matrix of unique molecular identifier (UMI) counts was then analyzed with Seurat V3 (*42*) for quality control, normalization, dimensional reduction, integration, clustering, and visualization. Quality control criteria were (i) total UMI count between 3000 and 30,000, (ii) minimal number of detected genes of >1000, (iii) mitochondrial gene percentage of <15%, and (iv) number of cells expressing a gene of >8. The count matrix was log normalized, and the top 2000 most variable genes were identified for dimensional reduction.

The count matrix was scaled, and the top 20 dimensions from the principal components analysis (PCA) were used for the UMAP. The top principal components were chosen using the Elbow plot method. Cell clusters were identified by the shared nearest neighbor method using the Louvain algorithm with the resolution of 1.2 and the top 20 PCA dimensions. Cell clusters of CD8^+^ T cells were annotated using known markers of T cells, and using SingleR to perform reference-based annotation, which compares the transcriptome of each cell cluster against various reference datasets (Human primary cell atlas, Database Immune Cell Expression Data, and Monaco immune data). For the final analysis, cell clusters were merged into six groups [naïve, cell cycle, blood, skin, Mucosal-Associated Invariant T(MAIT)-like, and memory-like].

### scTCR-seq data processing and analysis

Sequencing data were processed using the Cell Ranger VDJ pipeline (v7.1.0, 10x Genomics) with default settings. Briefly, Cell Ranger aligned TCR reads to the GRCh38 reference genome, determined consensus TCR sequences, and identified contigs and the corresponding CDR3 regions and V, D, J, and C genes. TCR clonotypes represented by CDR3 amino acid sequences and V gene usages were analyzed using scRepertoire (*43*).

### Statistical analysis

GraphPad Prism 10 (GraphPad Software Inc., CA, USA) was used for statistical analyses of flow cytometry data. Wilcoxon matched-pairs sign rank test was used for comparisons between blood and skin. Mann-Whitney *t* test was used for comparisons between two groups (patients with dengue versus healthy volunteers, primary versus secondary patients, and outpatients versus inpatients) and one-way analysis of variance (ANOVA) with Benjamini-Hochberg false discovery rate correction for multiple comparisons was used for comparison of more than two groups. Statistical analyses for the Olink data were performed by one-way ANOVA using Partek Genomic Suite Analysis version 7 software. For all analyses, *P* values are defined as follows: *P ≤ 0.05, **P ≤ 0.01, ***P ≤ 0.005, and ****P ≤ 0.0001.
