# Supplementary material for: Dengue infection elicits skin tissue-resident and circulating CD8+ T cells associated with protection from hospitalization
Source: Sci Adv. 2026 Mar 20;12(12):eaea7987. doi: 10.1126/sciadv.aea7987 (PMC13004022; doi:10.1126/sciadv.aea7987)
Supplement: Supplementary file 1 — Supplementary Methods Figs. S1 to S4 Tables S1 to S5 [file sciadv.aea7987_sm.pdf]

Science Advances
AAAS

Supplementary Materials for

Dengue infection elicits skin tissue-resident and circulating CD8$^+$ T cells associated with protection from hospitalization

Noor Zayanah Hamis *et al.*

Corresponding author: Laura Rivino, laura.rivino@bristol.ac.uk

Sci. Adv. **12**, eaea7987 (2026)
DOI: 10.1126/sciadv.aea7987

This PDF file includes:

Supplementary Methods
Figs. S1 to S4
Tables S1 to S5

Supplementary methods

DENV RNA qPCR from skin blister fluids

RNA was extracted from skin blister fluids using the Pathogen 200 protocol on the Roche MagNA Pure 24 Instrument according to the manufacturer's instructions. DENV serotype of infection was determined using the Center for Disease Control and Prevention (CDC) DENV-1–4 real-time RT-PCR (qRT-PCR) Assay singleplex protocol with the Vazyme HiScript III One Step qRT-PCR Probe 5X Master Mix Kit (Vazyme, Cat No: #Q611) on the Roche LightCycler 96. The primers and probes used are listed in **Table S3**.

Supplementary figures

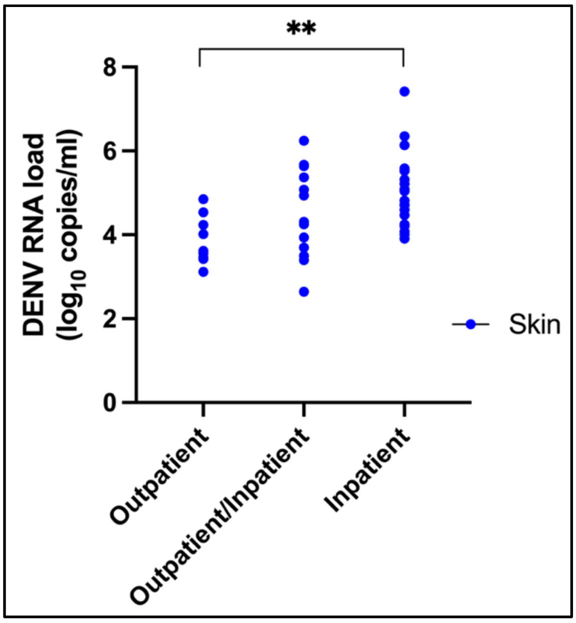

Figure S1. DENV viral load in skin blister fluids. DENV RNA was measured by qPCR in skin blister fluid from 47 patients at Visit 2. For this analysis we included samples with detectable RNAemia at Visit 1 (Table 1). Statistics were determined by Kruskal-Wallis test between hospitalisation statuses.

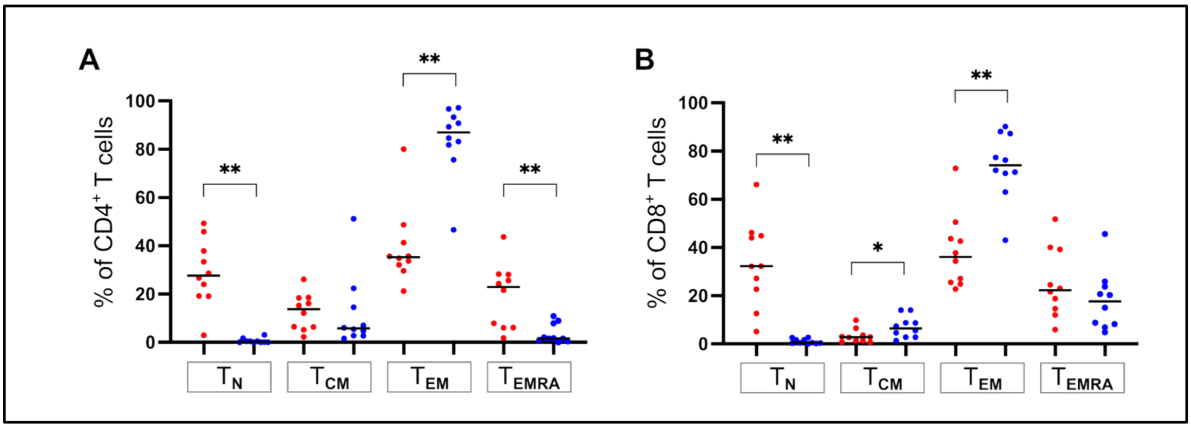

Figure S2. Frequencies of blood and skin T-cell subsets in healthy volunteers. T-cell subsets defined by CCR7 and CD45RA expression: T_N (naïve): CCR7+CD45RA+; T_{CM} (T central memory): CCR7+CD45RA-; T_{EM} (T effector memory): CCR7-CD45RA-; T_{EMRA} (T effector memory re-expressing CD45RA): CCR7-CD45RA+. Datapoints for blood and skin samples for each participant are shown respectively in red and blue. Statistics were determined by Wilcoxon matched-pairs sign rank test between blood and skin.

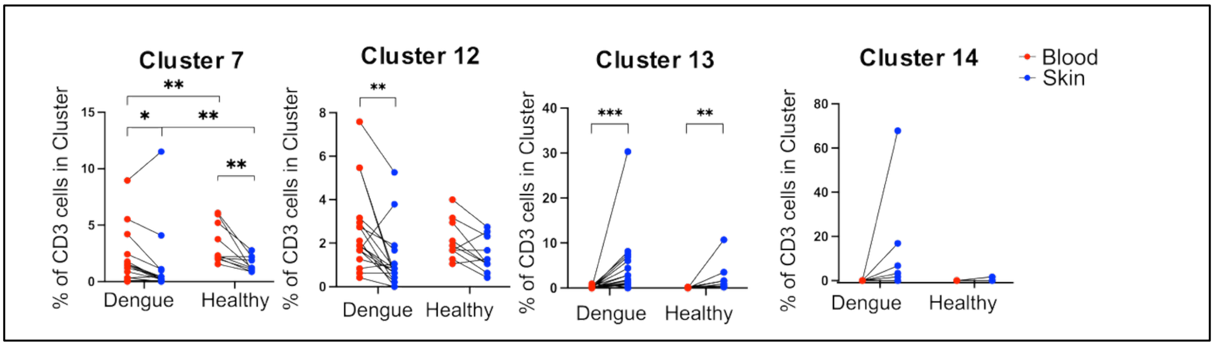

Figure S3. Skin and blood T-cells are largely distinct. Frequencies of cells within clusters defined by phenograph in Fig. 3 in the skin and blood of dengue patients and healthy volunteers. Statistics were calculated by Wilcoxon matched-pairs sign rank test between blood and skin, and Mann-Whitney t-test between healthy volunteers and patients.

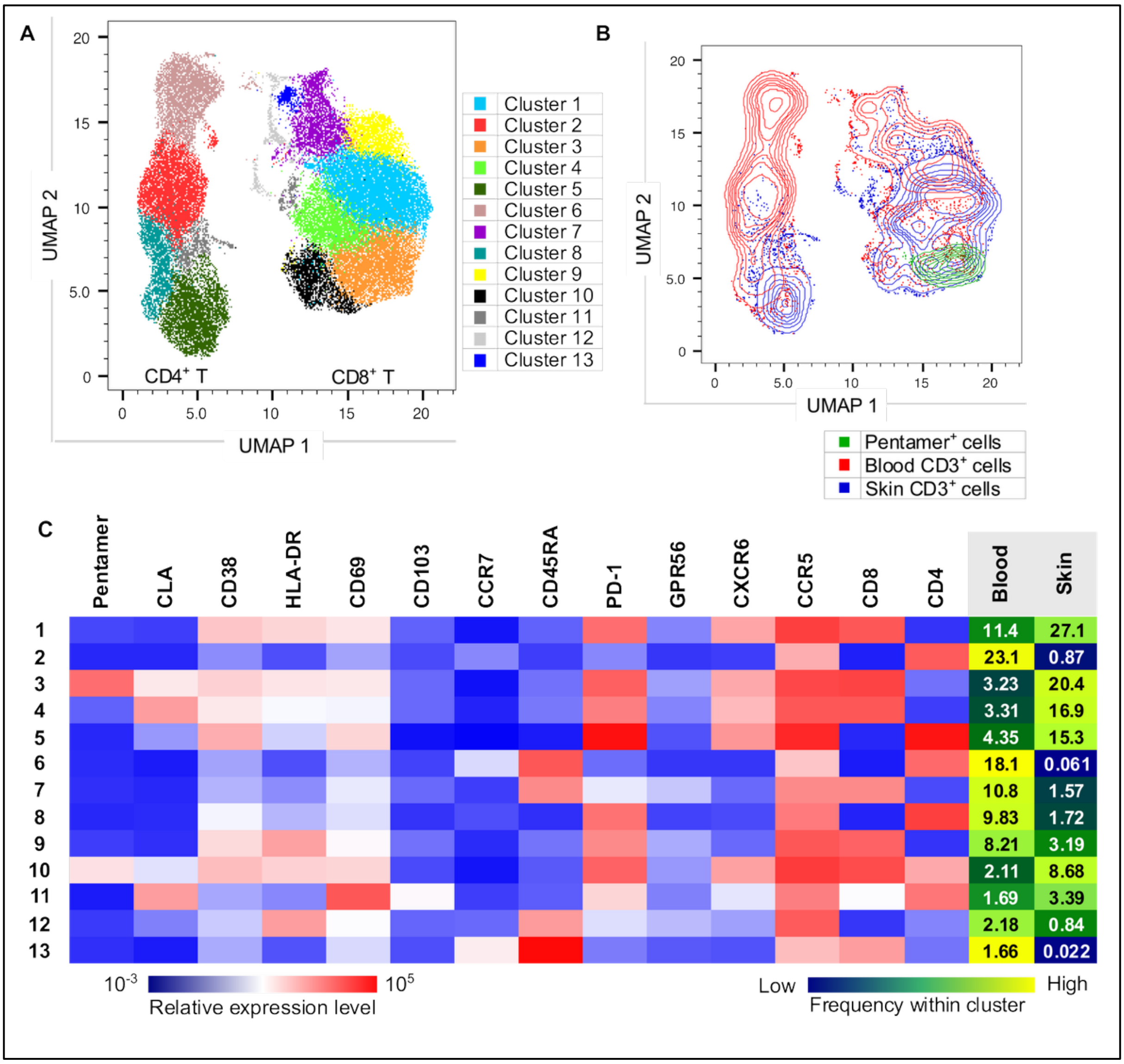

Figure S4. DENV pentamer+ CD8+ T-cells are enriched in the skin. (A) UMAP plot with phenograph clustering is shown for skin and blood T-cells of a dengue patient. **(B)** Skin (blue) and blood (red) CD3+ T cells, and manually-gated pentamer+ DENV-specific CD8+ T cells (green) cells are shown. **(C)** Mean fluorescence intensity (MFI) of the analysed markers for each cluster are displayed is shown in a heatmap. Frequencies of each phenograph cluster within the blood and skin are displayed in the respective columns.

Supplementary Tables

Table S1: Details for patients included in this study stratified by hospitalization status. Statistics were determined by Kruskal-Wallis test and Fisher’s exact test.

	**Outpatient (N=23)**	**Inpatient (N=31)**	**Outpatient/ Inpatient (N=20)**	**p-value**
**Age**				
**Mean ± SD**	36 ± 11	43 ± 13	38 ± 14	0.06
**Sex**				
**Male**	20 (87%)	24 (77%)	16 (80%)	
**Female**	3 (13%)	7 (23%)	4 (20%)	0.7
**2009 WHO Classification**				
**Severe Dengue**	1 (4%)	1 (3%)	1 (5%)	
**Dengue With Warning Signs**	10 (43%)	23 (74%)	11 (55%)	
**Dengue Without Warning Signs**	12 (52%)	7 (23%)	8 (40%)	0.15
**Serostatus**				
**Primary infection**	17 (74%)	13 (42%)	10 (50%)	
**Secondary infection**	6 (26%)	18 (58%)	10 (50%)	0.06
**Serotype**				
**DENV1**	0 (0%)	2 (6%)	0 (0%)	
**DENV2**	4 (17%)	22 (71%)	8 (40%)	
**DENV3**	10 (44%)	3 (10%)	6 (30%)	
**DENV4**	0 (0%)	0 (0%)	1 (5%)	***
**Unknown (<LOD)**	9 (39%)	4 (13%)	5 (25%)	0.0006

**Thrombocytopenia**				
**With**	10 (44%)	27 (87%)	16 (80%)	**
**Without**	13 (56%)	4 (13%)	4 (20%)	0.002
**Rash**				
**With**	7 (30%)	15 (48%)	6 (30%)	
**Without**	16 (70%)	16 (52%)	14 (70%)	0.29
**Highest hematocrit**				
**Mean ± SD**	45.7 ± 2.6	46.0 ± 4.6	45.4 ± 4.3	0.71
**Lowest platelet count**				
**Mean ± SD (x10^3/ µL)**	118.2 ± 42.2	51 ± 39.5	58.9 ± 46.1	**** <0.0001

Table S2: Details for the patients included in the ScRNA-seq analyses. Indicated are the number of activated $CD8^+$ T-cells sorted from each patient sample.

Patient	Days from fever onset	Age	Infection	Rash	Hospitalization	Bleeding	Blood- No. of $CD38^+$HLA-DR^+ CD8+ T cells	Skin- No. of CD38+HLA-DR+ CD8+ T cells
P1	10	29	Primary	Yes	Outpatient	No	1700	803
P2	7	26	Secondary	Yes	Outpatient/ Inpatient	Yes	1700	612
P3	8	31	Primary	No	Inpatient	Yes	2000	1510

Table S3: Primers and probes used for the Center for Disease Control and Prevention (CDC) DENV-1–4 RT-PCR Assay.

	Primers		Probes	
**DENV 1**	F	5’ CAA AAG GAA GTC GTG CAA TA 3’	D1-FAM-Probe	5’ /6-FAM/ CAT GTG GTT /ZEN/ GGG AGC ACG C /3IABkFQ/ 3’
	C	5’ CTG AGT GAA TTC TCT CTA CTG AAC C 3’		
**DENV 2**	F	5’ CAG GTT ATG GCA CTG TCA CGA T 3’	D2-HEX-Probe	5’ /HEX/ CTC TCC GAG /ZEN/ AAC AGG CCT CGA CTT CAA /3IABkFQ/ 3’
	C	5’ CCA TCT GCA GCA ACA CCA TCT C 3’		
**DENV 3**	F	5’ GGA CTG GAC ACA CGC ACT CA 3’	D3-TexRd-Probe	5’ /TexRd-XN/ ACC TGG ATG TCG GCT GAA GGA GCT TG /3IAbRQSp/ 3’
	C	5’ CAT GTC TCT ACC TTC TCG ACT TGT CT 3’		
**DENV 4**	F	5’ TTG TCC TAA TGA TGC TGG TCG 3’	D4 Cy5-Probe	5’ /Cy5/ TTC CTA CTC /TAO/ CTA CGC ATC GCA TTC CG /3IAbRQSp/ 3’
	C	5’ TCC ACC TGA GAC TCC TTC CA 3’		

Table S4: Flow cytometry antibodies used.

	Antibody	Clone	Brand & Catalogue number	Staining volume (µL in 50µL)
1	BUV737 Mouse Anti-Human CD279 (PD-1)	EH12.1	BD Horizon, 612791	3
2	BUV395 Mouse Anti-Human CD45	HI30	BD Horizon, 563792	3
3	V500 Mouse Anti-Human CD3	UCHT1	BD Horizon, 561416	3
4	BV711 Mouse Anti-Human CD38	HIT2	BD Horizon, 563965	2.5
5	BV650 Mouse Anti-Human CD314 (NKG2D)	1D11	BD Horizon, 563408	5
6	BUV737 Mouse Anti-Human CD195 (CCR5)	2D7/CCR5	BD Horizon, 565293	5
7	APC-Cy7 Mouse Anti-Human CD8	SK1	BD Pharmingen, 557834	3
8	Alexa Fluor® 700 Mouse anti-Human CD197 (CCR7)	150503	BD Pharmingen, 561143	3

9	PE Mouse Anti-Human CD45RO	UCHL1	BD Pharmingen, 555493	3
10	PE-Cy7 Mouse Anti-Human HLA-DR	L243	BD, 335795	2.5
11	Brilliant Violet 785 anti-human CD69	FN50	Biolegend, 310932	5
12	Brilliant Violet 650™ anti-human HLA-DR	L243	Biolegend, 307650	1.3
13	BV605 Mouse Anti-Human CD45RA	HI100	Biolegend, 562886	0.5
14	FITC anti-human/mouse Cutaneous Lymphocyte Antigen (CLA)	HECA-452	Biolegend, 321306	10
15	PE/Cyanine7 anti-human GPR56	CG4	Biolegend, 358206	2.5
16	PE/Dazzle™ 594 anti-human CD4 Antibody	A161A1	Biolegend, 357412	1
17	Brilliant Violet 421™ anti-human CD279 (PD-1) Antibody	EH12.2H7	Biolegend, 329920	5

18	Alexa Fluor® 647 anti-human CD186 (CXCR6) Antibody	K041E5	Biolegend, 356008	2
19	Brilliant Violet 421™ anti-human Ki-67 Antibody	Ki-67	Biolegend, 350506	5
20	CD103 (Integrin alpha E) Monoclonal Antibody PerCP-eFluor710	Ber-ACT8	eBioscience, 46-1037-42	2.5
21	APC anti-human Granzyme B Antibody	GB11	Invitrogen, GRB05	5
22	LIVE/DEAD™ Fixable Blue Dead Cell Stain Kit, for UV excitation		Life Technologies, L23105	1:1000

Table S5: List of PE-conjugated peptide-HLA pentamers purchased from ProImmune Limited and the sequence for each peptide are shown.

	**Sequence**	**Epitope**	**Staining volume (µL in 50µL)**
**DENV 1, HLA-A*11:01**	GTSGSPIVNR	NS3 1608-1617	10µL each

	ATYGWNLVK	NS5 2610-2618	
**DENV 2, HLA-A*11:01**	GTSGSPIIDK	NS3 1608-1617	
	STYGWNLVR	NS5 2610-2618	
**DENV 3, HLA-A*11:01**	GTSGSPIINR	NS3 1608-1617	
	STYGWNIVK	NS5 2610-2618	
**DENV 4, HLA-A*11:01**	GTSGSPIINR	NS3 1608-1617	
